# Anticancer activity of milk fat rich in conjugated linoleic acid against Ehrlich ascites carcinoma cells in female Swiss albino mice

**DOI:** 10.14202/vetworld.2021.696-708

**Published:** 2021-03-20

**Authors:** Abdelrahman M. Abd El-Gawad, Diea G. Abo El-Hassan, Ahmed M. Aboul-Enein, Sherein S. Abdelgayed, Salwa A. Aly, Gamal Esmat, Amr A. Mostafa, Mohamed H. Bakr, Rida A. Ali, Mahmoud A. Ayoub

**Affiliations:** 1 Department of Animal Production, Faculty of Agriculture, Cairo University, Giza, Egypt; 2 Department of Medicine and Infectious Diseases, Faculty of Veterinary Medicine, Cairo University, Giza, Egypt; 3 Department of Biochemistry, Faculty of Agriculture, Cairo University, Giza, Egypt; 4 Department of Pathology, Faculty of Veterinary Medicine, Cairo University, Giza, Egypt; 5 Department of Food Hygiene, Faculty of Veterinary Medicine, Cairo University, Giza, Egypt; 6 Department of Hepatogastroenterology and Infectious Diseases, Faculty of Medicine, Cairo University, Giza, Egypt; 7 Department of Clinical and Chemical Pathology, National Cancer Institute, Cairo University, Giza, Egypt

**Keywords:** anticancer activity, conjugated linoleic acid, Ehrlich ascites carcinoma % increase in life span, mean survival time, tumor transplanted mice

## Abstract

**Background and Aim:**

The major conjugated linoleic acid (CLA) isomers have anticancer effect, especially breast cancer cells, inhibits cell growth and induces cell death. Also, CLA has several health benefits *in vivo*, including antiatherogenesis, antiobesity, and modulation of immune function. The present study aimed to assess the safety and anticancer effects of milk fat CLA against *in vivo* Ehrlich ascites carcinoma (EAC) in female Swiss albino mice. This was based on acute toxicity study, detection of the tumor growth, life span of EAC bearing hosts, and simultaneous alterations in the hematological, biochemical, and histopathological profiles.

**Materials and Methods:**

One hundred and fifty adult female mice were equally divided into five groups. Groups (1-2) were normal controls, and Groups (3-5) were tumor transplanted mice (TTM) inoculated intraperitoneally with EAC cells (2×10^6^/0.2 mL). Group (3) was (TTM positive control). Group (4) TTM fed orally on balanced diet supplemented with milk fat CLA (40 mg CLA/kg body weight). Group (5) TTM fed orally on balanced diet supplemented with the same level of CLA 28 days before tumor cells inoculation. Blood samples and specimens from liver and kidney were collected from each group. The effect of milk fat CLA on the growth of tumor, life span of TTM, and simultaneous alterations in the hematological, biochemical, and histopathological profiles were examined.

**Results:**

For CLA treated TTM, significant decrease in tumor weight, ascetic volume, viable Ehrlich cells accompanied with increase in life span were observed. Hematological and biochemical profiles reverted to more or less normal levels and histopathology showed minimal effects.

**Conclusion:**

The present study proved the safety and anticancer efficiency of milk fat CLA and provides a scientific basis for its medicinal use as anticancer attributable to the additive or synergistic effects of its isomers.

## Introduction

Cancer is a category of malignant illnesses that are caused by rapid and uncontrolled formation of abnormal cells which may lump forming tumor or proliferate and imitate abnormal growth at other sites in the body [1]. It is a devastating disease with tremendous negative implications for the personal, health-care economics, and social levels [2]. Statistically, it is the second major cause of human death after cardiovascular diseases worldwide and is responsible for the deaths of 9.6 million person in 2018 [3]. About 14.1 million cancer cases were diagnosed all over the world in 2012; of these, 52.5% were in men and 47.5% in women, and will rise up to 21.7 million by 2030 [4]. It causes about 13% of all human deaths and according to the American Cancer Society around 7.6 million people die every year from cancer [5]. Several methods are used for the treatment of cancer, such as chemotherapy, radiotherapy, and surgery, the chemotherapy is now considered as an efficient method for treatment. Success of cancer chemotherapy is limited by drugs that have hepatotoxic, nephrotoxic, cardiotoxic, myelosuppressive, multidrug resistance, and other side effects [6] and have become serious medical problems [7]. Recent strategies for cancer prevention depend on the modification of lifestyle such as diet, some dietary elements such as conjugated linoleic acid (CLA) can minimize the cancer risk [8]. Therefore, many extensive studies have been devoted recently to search for natural preventive and therapeutic approaches with antitumor, antioxidant, and anti-inflammatory potential can treat various kinds of diseases with less side effects [9]. Milk and its products are considered as important sources of energy and bioactive substances positively associated with human health [10].

Tumor cell line, Ehrlich ascites carcinoma (EAC); is an undifferentiated carcinoma, not have a tumor-specific transplantation antigen, of 100% malignancy, highly transplantable, very rapid proliferative, and shorter lifespan [11]. It is mostly used to assess the anticancer activity of different agents [12]. This cell line is more sensitive to chemotherapy and used in determining whether the tumor is responding to therapy or not and has similarity with human tumors due to its undifferentiation and rapid growth rate nature [13].

More than 28 different geometrical and positional isomers of linoleic acid conjugated with a double bond system are defined as CLA. Many biological impacts of CLA until now are imputed to two major isomers, namely, cis-9 (c9), trans-11 (t11) and trans-10 (t10), cis-12 (c12) constitute about 90% and 10% of the total CLA isomers. These isomers are synthesized through a biohydrogenation process by the ruminal bacteria in ruminants, or through bioconversion in mammary gland [14]. The isomers of CLA are active biological molecules, have protective effects against several diseases include atherosclerosis, obesity, osteoporosis, diabetes, cancer, and certain chronic inflammatory afflictions [15]. Anti-carcinogenic effects have been observed in all cancer types, with doses varying between 55 mg and 3.5 g CLA/day [16]. Milk and its products represent main source of CLA in the human diet, with nearly 70% of the daily requirement, and an average intake of 650 mg/day. That intake value is insufficient for achieving beneficial effects on human health. Various technological alternatives in the field of food technology are exploring ways to produce milk and dairy product rich in CLA [17]. Several studies on human nutrition recorded that daily safe requirement of CLA ranged between 3 and 6 g, although some researches mentioned that administration more than 3.4 g CLA/day has no beneficial action [18].

Loss of endogenous estrogen production after menopause increases the risk of osteoporosis, cardiovascular diseases, and obesity [19]. Estrogen surrogate therapy effectively reduces and/or prevents these health issues in postmenopausal women [20]. At the same time, exogenous estrogen administration increases the risk of endometrial hyperplasia and breast cancer [21], so there is great interest in natural alternatives to estrogen therapy to avoid postmenopausal hazard on women’s health. In postmenopausal women, CLA can inhibit the estrogen receptor (ER) signaling in human endometrial and breast cancer cells [22], acting as an estrogen antagonist through the inhibition of ER alpha (ERa)-mediated responses. Bocca *et al*. [23] reported the strong anticancer action of CLA through the ER signaling inhibition. Amaru *et al*. [24] concluded that CLA effectively reduces breast cancer risk by inhibiting breast tumor initiation, promotion, and progression. Concerning colorectal cancer, CLA intake succeeded in achieving 30% reduction and showed significant role in preventing testicular cancer [25].

From this viewpoint, this study was performed to assess the efficacy of milk fat rich in CLA on modulating cancer induced by Ehrlich cells in female Swiss albino mice to enhance a natural tumor therapy. To achieve this goal, several parameters include cell growth inhibition, volume of the ascetic fluid, and mean survival time (MST) of tumor transplanted mice (TTM); in addition, restoration of the hematological, biochemical, and histopathological alterations was studied.

## Materials and Methods

### Ethical approval

This study protocol was approved by the Institutional Animal Care and Use Committee, the Ethics Committee of the Faculty of Veterinary Medicine, Cairo University, Giza, Egypt (Approval No. VetCU10102019088).

### Study period and location

This study was conducted from September 2019 to March 2020 at the Laboratory of Faculty of Agriculture and Veterinary Medicine, Cairo University, Egypt.

### Milk fat rich in CLA

The milk fat rich in CLA was produced from Holstein-Friesian Cows fed on diet contained protected fat, high in unsaturated fatty acids. The CLA percentage of the produced milk fat was 2% of the fatty acids, while the percentage of CLA in normal cow milk fat ranged between 0.3% and 0.4%. The cow milk fat rich in CLA was supplemented to the normal balanced diet of mice (10% w/w) and used in feeding the experimental female mice orally at a level of 50 mg (containing 1 mg CLA) per mice, 40 mg CLA/kg body weight (BW), daily.

### Experimental animals

One hundred and fifty adultfemale Swiss albino mice (23-25 g) from the Laboratory Animal Farm in Helwan, Egypt, were housed in plastic mesh cages under strict standard hygienic measures. Female mice selected for this study because EAC is grown and divided in female peritoneal mice and not in male, due this tumor is working on female hormones. The mice were adapted to the laboratory circumstance, 28 days before the experiment onset. The mice were fed on a balanced ration with a good source of water *ad libitum*, during the acclimatization and experimental periods and were exposed to 12 h of light/ dark cycle during the study.

### Acute toxicity study

The acute oral toxicity investigation of the milk fat enriched CLA was performed in six adult female Swiss albino mice, two mice per treatment dose, at three increasing oral doses 50, 100, and 200 mg of the tested milk fat rich in CLA equivalent 40, 80, and 160 mg CLA/kg BW, respectively. Following treatment, the mice were observed for 48 h for any mortality or behavioral changes [26]. Adding different concentrations of milk fat to ration did not produce any mortality in any of the tested dose levels, this encouraged us to perform further assessments using the dose level of 50 mg milk fat (40 mg CLA/kg BW).

### Ascitic tumor induction

The tumor cell line, EAC, was kindly obtained from the National Cancer Institute, Cairo, Egypt, and propagated intraperitonially (i/p) in Swiss albino mice [27]. Viable tumor cells were detected using Trypan blue stain and counted by hemocytometer. The ascitic fluid was diluted in normal saline to obtain tumor cell suspension of 10×10^6^ cells/mL. From this stock suspension, 0.2 mL (2×10^6^ cells/mice) was inoculated i/p to induce ascitic tumor.

### Experimental design

One hundred and fifty adult female Swiss albino mice, about 25 g each, were equally divided into five groups for anticancer activity evaluations. Group 1 (G1) was kept as normal control; feed on normal balanced ration without tumor cell induction. Group 2 (G2) feed on normal balanced ration supplemented with 50 mg milk fat, 1 mg CLA/mice, (40 mg CLA/kg BW) by oral route daily without tumor cell inoculation. Group 3 (G3) was positive TTM, inoculated i/p by EAC cells (2×10^6^ cells/mouse) and fed on normal balanced ration. Group 4 (G4) was inoculated (i/p) by EAC cells (2×10^6^ cells/mouse), after 24 h of inoculation, the mice were treated with 50 mg milk fat, contained 1 mg CLA/mice (40 mg CLA/kg BW) by oral route daily. Group 5 (G5) was inoculated (i/p) by EAC cells (2×10^6^ cells/mouse), fed with 50 mg milk fat, 1 mg CLA/mice, (40 mg CLA/kg BW) by oral route daily, 28 days pre inoculation. The oral administration of the treatments was done in the form of paste, representing 10% of the normal mice ration, daily. After 16 days of tumor induction, ten mice of each group were fasted for 18 h, then anesthetized with diethyl ether and sacrificed. The ascitic fluid was collected and tumor growth was assessed. Percent of tumor growth inhibition was calculated by comparing the tumor cells count in the ascitic fluid of treated groups and the TTM positive control group (G3). Tumor cell growth in peritoneal fluids of the normal control group (G1) was taken as 100% cell growth to assess the anticancer action of the milk fat CLA.

For histopathological examination, liver and kidneys were harvested. The twenty remaining mice were used for estimating the MST and percent increase in life span (% ILS). BW of mice, ascitic fluid volume, hematological parameters, red blood cells (RBCs), hemoglobin (Hb), white blood cells (WBCs), differential count, and biochemical parameters was evaluated.

### Evaluation of the anticancer activity

The anticancer activity of milk fat CLA was assessed through definitive parameters include the MST, % ILS, tumor weight and volume, and restoring of the hematological, biochemical, and histopathological alterations [28].

### Life span

Survival time for TTM and effect of milk fat CLA on tumor growth was observed by MST and % ILS [29].

### MST

Twenty mice in each group were monitored by 5 weeks. Survival days of each animal from the day of tumor induction were counted, and spontaneous death of mice was considered the endpoint of experiments. The calculation of MST was calculated according to the following equation:


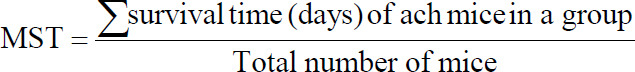


### % ILS

The effect of milk fat CLA on % ILS of the animals was calculated by comparing the survival time for treated group with that of control group using the following equation:


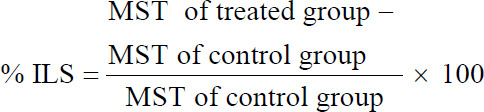


### Tumor weight and tumor volume

For the determination of tumor weight, ten mice from each group were sacrificed on day 16 of inoculation; the weight was recorded before and after the collection of the ascitic fluid. The difference in weight before and after gives the tumor weight and is expressed in grams. Tumor volume was determined by measuring the ascitic fluid volume using a graduated centrifuge tube [30].

### Counting of viable tumor cells

The ascitic fluid was collected using WBCs diluting pipette and diluted up to 100 times with phosphate-buffered saline. The viable cell counting was performed in 64 squares on Neubauer’s chamber using a drop of the prepared dilution. Trypan blue stain (0.4% in normal saline) was used to assess the viable cells, viable cells did not take the color of the dyes [30].

### Blood sampling

From the sacrificed mice, 2 mL of blood were taken per mouse and placed on previously two identified microtubes; one with ethylenediamine tetra-acetic acid (EDTA) 10% as anticoagulant used to measure hematological parameters, and the second one without anticoagulant for separation of serum was used to measure the biochemical parameters [31].

### Hematological parameters

Blood parameters were performed according to Santos *et al*. [32]. Blood on EDTA was used for RBC counts, estimation of Hb levels, and WBC counts. Differential count of WBCs was determined using blood smears stained with Giemsa stain.

### Biochemical parameters

Biomarkers of liver function include serum alanine aminotransferase (ALT), aspartate aminotransferase (AST), and alkaline phosphatase (ALP) activities in addition to serum creatinine, a biochemical analysis for renal function evaluation, were measured and interpreted, according to Tietz [33]. Total protein was measured by means of Biuret reagent and albumin was determined using the modified Bromocresol green, according to Moreira *et al*. [34]. Globulins were measured by subtracting of albumin from total serum proteins and albumin/globulin (A/G) ratio was calculated from dividing albumin value on globulin value for each sample. Liver and renal function tests were performed to assess functioning of the liver and kidneys after milk fat CLA administration. Total antioxidant capacity (TAC) was estimated, according to Lalithadevi *et al*. [35]. The previous biochemical parameters were measured colorimetrically using bio-diagnostic kits.

### Histopathological examination

The treated mice and their controls were anesthetized with diethyl ether and immediately sacrificed, quickly dissected; liver and kidney were removed and fixed in Bouin’s fluid. After 24 h, tissues were rinsed 3 times in 70% ethanol, dehydrated using a graded ethanol series and then embedded in paraffin wax. Paraffin thick slices, 5 mm, were stained with hematoxylin and eosin and examined under light microscope and photographed using a digital microscope (Olympus BX50, Japan) [36].

### Statistical analysis

The experimental results were expressed as the mean±standard error of the mean. The significant difference between the groups was statistically determined by one-way analysis of variance. Data were accepted as statistically significant considering the difference at p<0.05.

## Results and Discussion

Cancer remains a major health problem causing mortality despite novel findings and several new anticancer drugs, its incidence is increasing with an annual rate of 1.2% [37]. Improvement in cancer treating strategies will result in prolonged survival of patients; however, there is a growing need for additional means of cancer management in both palliative and curative treatments [38].

### Acute toxicity of milk fat CLA

Milk fat CLA was safe at doses as high as 3 mg/mouse daily (160 mg CLA/kg BW) by oral route. The behavior of the mice was monitored, every 4 h, for a period of 48 h. Milk fat CLA supplemented diet did not induce mortality, behavioral changes, locomotor ataxia, diarrhea, or weight loss in mice during the 48-h observation period. Furthermore, feed and water intake did not differ among the groups of study. Dilzer and Park [15] reported similar findings.

### Anticancer activity

#### Effect on MST and % ILS

**Figure-1:**
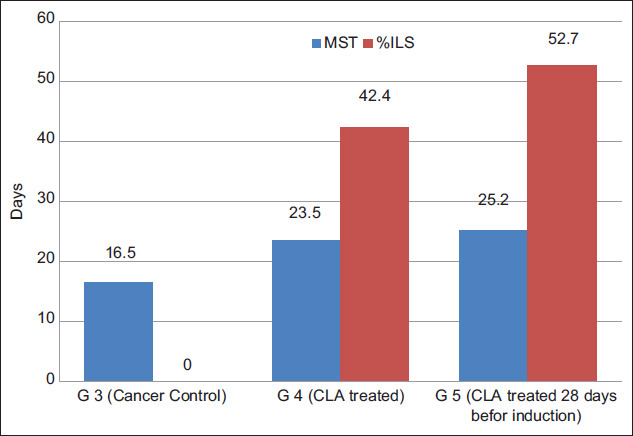
Mean survival time/day and percent increase in life span of tumor transplanted mice treated with milk fat rich in conjugated linoleic acid.

Effect of milk fat CLA on life span, MST and % ILS, of TTM was investigated (Table-1 and Figure-1). In TTM positive control group (G3), the MST was 16.5 days while it significantly (p<0.05) increased to 23.5 and 25.2 days in G4 and G5 fed 1 mg milk fat CLA (40 mg CLA/kg BW.) daily 28 days post- and pre-tumor cells inoculation, respectively. The administration of CLA at a dose of 40 mg/kg BW, orally significantly (p<0.05) increased the % ILS values to 42.4% and 52.7% in the treated groups (G4) and (G5), respectively, when compared with the TTM, positive control group (G3). The results of survival analysis revealed that MST and ILS% were significantly increased in milk fat CLA treated mice (Groups 4 and 5).

**Table-1:** Effect of daily oral administration of milk fat rich in CLA on MST and % ILS in EAC inoculated mice.

Groups	Treatment	MST/days	% ILS
Group 3	Positive control (TTM)	16.5±0.19	-
Group 4*	TTM feed on 50 mg milk fat/mouse (1 mg CLA) daily post-inoculation.	23.5±0.26*	42.42*
Group 5*	TTM feed on 50 mg milk fat/mouse (1 mg CLA) daily 28 days pre-inoculation.	25.2±0.35*	52.73*

The values expressed as mean±SE, TTM=Tumor transplanted mice with EAC cells,

* Significant at (p<0.05) as compared with positive control group (Group 3, TTM). CLA=Conjugated linoleic acid, MST=Mean survival time, EAC=Ehrlich ascites carcinoma, % ILS=Percent increase in life span, TTM=Tumor transplanted mice, MST=Mean survival time

### Effect on tumor weight and tumor volume

**Figure-2:**
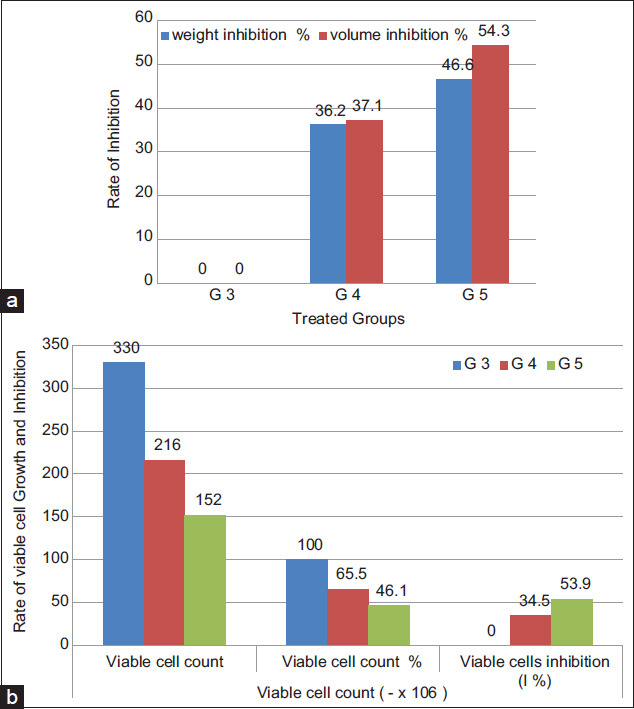
Tumor growth rate in tumor transplanted mice treated with milk fat rich in conjugated linoleic acid: (a) Weight and volume inhibition percent, (b) viable cell count and percentage of viable cell inhibition.

Milk fat CLA supplement leads to significant (p<0.05) reduction in the weight and volume of tumor in TTM treated groups (Table-2 and Figure-2a). Tumor weight of TTM control group (G3) was found to be 5.8 g and that of milk fat CLA treated mice G4 and G5 at a dose of 40 mg CLA/kg BW, was 3.7 and 3.1 g with weight growth inhibition 36.2 and 46.6%, respectively. The ascitic fluid volume of the TTM control group was 7 mL, and it was significantly (p<0.05) reduced to 4.3 and 3.2 mL with a volume inhibition 37.1 and 54.3% in treated mice groups G4 and G5, respectively.

**Table-2:** Effect of daily oral administration of milk fat rich in CLA on tumor growth in EAC inoculated mice.

Groups	Treatment	Mean body weights	Tumor growth			

			W (g)	WI (%)	V ( mL)	VI (%)
Group 1	Normal control (mice feed on balanced ration without inoculation)	24.9±0.3	-	-	-	-
Group 2	Mice feed on 50 mg milk fat/mouse (1 mg CLA) daily for 28 days without inoculation	25.0±0.3	-	-	-	-
Group 3	Positive control (TTM) feed on balanced ration	30.7±0.1	5.8±0.1*	100.0	7.0±0.4*	100.0
Group 4	TTM feed on 50 mg milk fat/mouse (1 mg CLA) daily post-inoculation.	28.6±0.1	3.7±0.2**	36.2**	4.4±0.2**	37.1**
Group 5	TTM feed on 50 mg milk fat/mouse (1 mg CLA) daily 28 days pre-inoculation.	28.1±0.1	3.1±0.2**	46.6**	3.2±0.1**	54.3**

The values expressed as mean±SE, W=Weight, WI=Weight inhibition, V=Volume, VI=Volume inhibition,

* Significant at P<0.05 as compared with normal control group (Group 1),

** Significant at P<0.05 as compared with EAC group (Group 3). CLA=Conjugated linoleic acid, EAC=Ehrlich ascites carcinoma, TTM=Tumor transplanted mice

### Effect on viable cell count (cells×0^6^/mL)

As shown in Table-3 and Figure-2b, the mean viable cell count of TTM positive control group (G3) was 330±0.2 and significantly (p<0.05) decreased to 216±1 and 152±9 in treated mice G4 and G5, at dose of 40 mg/kg BW, respectively. The viable cell reduction in both treated groups was 65.5% and 46.1% while tumor cell inhibition was 34.5% and 53.9%, respectively. The antitumor nature of milk fat CLA was evidenced by the significant inhibition in tumor weight and ascitic volume and the significant reduction in viable tumor cell count, in milk fat CLA treated mice as compared to the TTM positive control group (G3).

**Table-3:** Effect of daily oral administration of milk fat rich in CLA on viable cell count.

Groups	Viable cell count (×10^6^)		

	Viable cell count (Number of tumor cells)	Viable cell count % (Tumor cell growth %)	Viable cells inhibition (I%) (Tumor cells inhibition %)
Group 3	330±2	100.0	100.0
Group 4	216±1*	65.5*	34.5*
Group 5	152±9*	46.1*	53.9*

The values expressed as mean±SE.

* Significant at (p*<*0*.*05) as compared with positive control group (Group 3, TTM). CLA=Conjugated linoleic acid, TTM=Tumor transplanted mice

**Figure-3:**
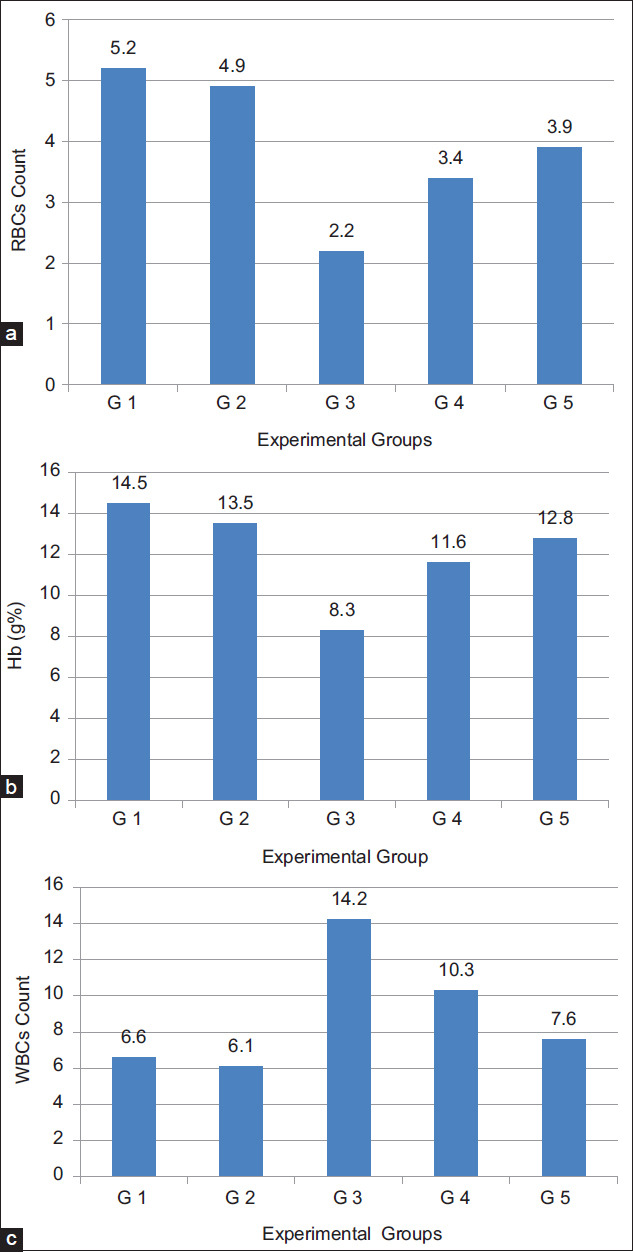
Effect of daily oral administration of milk fat rich in conjugated linoleic acid on: (a) Red blood cells count, (b) hemoglobin content, and (c) white blood cells count.

EAC is one of the commonly used experimental breast tumor that derived from spontaneous mouse adenocarcinoma that characterized by ascites [28]. In cancer, the increased volume of ascitic fluid indicates tumor growth; hence, decrease in ascitic fluid volume and arresting the tumor growth increase the life span of animals [39]. The obtained results *in vivo* supported this hypothesis as i/p inoculation of EAC cells into mice significantly increased the number of life EAC cells and subsequent ascites. Oral administration of 50 mg milk fat rich in CLA per mice (40 mg CLA/kg live BW) to TTM treated groups (G4 and G5) significantly (p<0.05) enhanced the MST and increased the life span of the inducted mice (Figure-1), significantly decreased tumor weight and volume (Figure-2a), number and percent of life EAC cells (Figure-2b) as well as WBCs count (Figure-3c), as compared to the TTM positive control (G3). Reddy *et al*. [38] reported that a 25% increase in the life span of ascites bearing animals is a significant indication of drug activity. The observations in the present experiment indicated the effectiveness of milk fat CLA on EAC cells and its role in the delay of cell division, thereby suggesting the reduction in EAC volume and increased survival time in mice. This meets the steadfast criteria, prolongation of life span and decrease in WBC, for judging the potency of any anticancer agent [30,38,39].

### Hematological parameters

**Figure-4:**
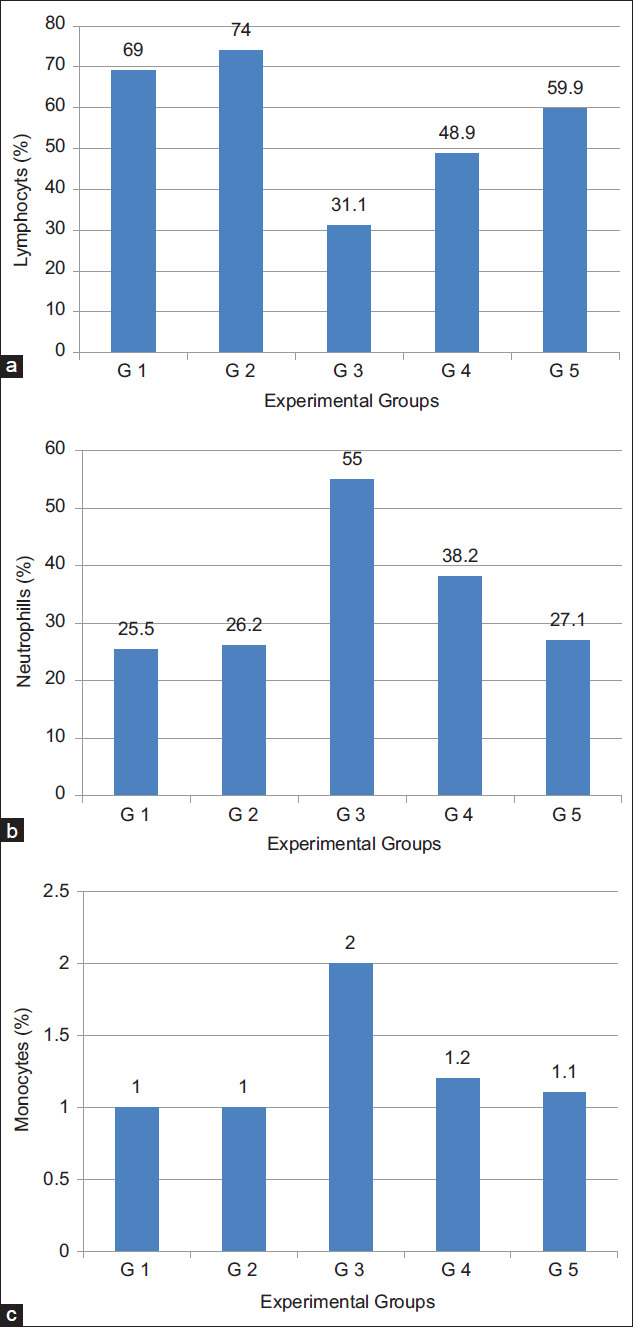
Effect of daily oral administration of milk fat rich in conjugated linoleic acid on: (a) Lymphocytes, (b) neutrophils, and (c) monocytes %.

The results in Table-4, Figures-3 and 4 showed that; on the 16^th^ day after tumor cells inoculation, the hematological parameters of TTM positive control group (G3) were significantly (p<0.05) altered, relative to the normal group (G1). The RBCs count (Figure-3a) and Hb (Figure-3b) were significantly (p<0.05) decreased to 2.2×10^6^ cells/mm^3^ and 8.3 g%, respectively, while WBCs count (Figure-3c) was significantly (p<0.05) increased to 14.2×10^3^ cells/mm^3^. In addition, the differential count of WBCs showed significant decrease (31.1%) in the percentage of lymphocytes (Figure-4a) while the percentage of neutrophils (Figure-4b) and monocytes (Figure-4c) significantly increased (55.0% and 2.0%), respectively. Significantly (p<0.05) restoring all the hematological alterations, in the treated groups (G4) and (G5) to near normal values, after oral administration of milk fat CLA at the same time period.

**Table-4:** Effect of daily oral administration of milk fat rich in CLA on hematological parameters in EAC inoculated Mice.

Groups	RBC count (10^6^cells/mm^3^)	Hb (g %)	WBC count (10^3^cells/mm^3^)	Lymphocytes (%)	Neutrophils (%)	Monocytes (%)
Group 1	5.2±0.1	14.5±0.7	6.6±0.1	69±0.9	25.5±0.8	1±0.3
Group 2	4.9±0.1	13.5±0.3	6.1±0.1	74±0.2	26.2±0.4	1±0.5
Group 3	2.2±0.1*	8.3±0.2*	14.2±0.2*	31.1±1.0*	55±1.9*	2±0.6*
Group 4	3.4±0.1**	11.6±0.2**	10.3±0.20**	48.9±2.1**	38.2±0.9**	1.2±0.3**
Group 5	3.9±0.2**	12.8±0.3**	7.6±0.15**	59.9±1.5**	27.1±0.7**	1.1±0.1**

The values expressed as mean±SE,

* Significant at P<0.05 as compared with normal control group (Group 1),

** Significant at P<0.05 as compared with EAC group (Group 3). SE=Standard error, CLA=Conjugated linoleic acid, EAC=Ehrlich ascites carcinoma

Anemia in TTM or during chemotherapy protocols, mainly due to reduction in RBC or hemoglobin percentage as a result of iron deficiency or myelosuppression [40]. Restoration of hemoglobin content and maintenance for the nearly normal values of RBCs and WBCs count of the treated mice groups (G4 and G5) after 16 days of tumor induction clearly indicates that milk fat CLA was able to reverse the alteration in the hematological parameters consequent to tumor inoculation and possesses a protective action on the hemopoietic system, suggesting its anticancer nature without inducing myelotoxicity.

Lymphocyte count significantly increased in the TTM treated groups (G4 and G5) after administration of milk fat CLA demonstrating that, CLA has the potentials of improving the cellular immune system and its effectiveness in treatment of disease conditions caused by lymphocytopenia in mammals [41].

### Biochemical parameters

#### Liver function

**Figure-5:**
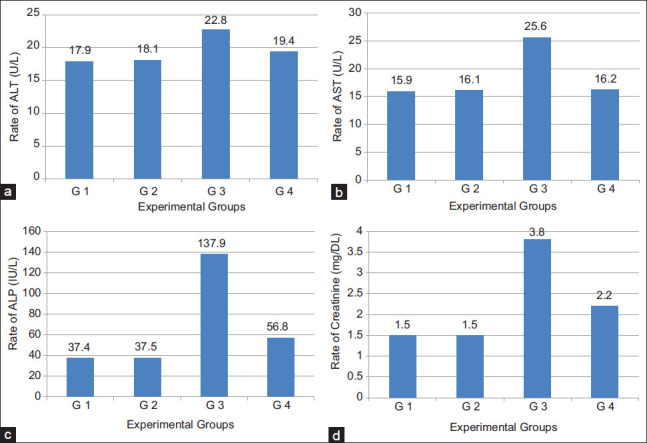
(a-d) Liver and kidney function tests in sera of Ehrlich ascites carcinoma inoculated mice after oral administration of milk fat rich in conjugated linoleic acid.

In respect to liver functions, transaminases and ALP levels were determined in the sera of the tested mice groups. Results, as shown in Table-5 and Figure-5, proved that supplementation of diet with milk fat CLA with level of 40 mg CLA/kg live BW did not affect transaminase, ALT and AST enzyme, activities in the sera of the tested normal animal group (G2). Induction of cancer into animals (G3), severely affect transaminases activity in its sera. The activities of both ALT (Figure-5a) and AST (Figure-5b) were significantly (p<0.05) elevated from 17.87 to 22.78 IU/L and from 15.89 to 25.61 IU/L, respectively. When TTM fed on diet supplemented with 40 mg CLA/kg live BW (Group G4), the elevation in transaminase activities significantly (p<0.05) lowered than that in TTM (G3). The enzyme activity levels reached nearly the normal levels as compared to Groups G1 and G2 but the levels were slightly still high (19.40±1.3 and 16.20, respectively). This means that CLA is highly effective for redressing disturbances in serum transaminases by cancer transplantation in experimental mice. The results of ALP activity in sera of the previous examined groups, showed similar trend as transaminases (Figure-5c). The levels were significantly (p<0.05) elevated from 37.38 to 137.92 IU/L by induction of tumor cells (G3) then, significantly reduced to 56.79 IU/L when the diet was supplement with 40 mg CLA/kg live BW (G4). However, the addition of milk fat CLA into the diet as supplement at a level of 40 mg CLA/kg BW greatly improved the negative effect of the cancer induction in animals.

**Table-5:** Activities of ALT, AST, ALP, Creatinine, and TAC in EAC inoculated mice sera after oral administration of milk fat rich in CLA.

Groups	Treatment	ALT (IU/L)	AST (IU/L)	ALP (IU/L)	Creatinine (mg/DL)	TAC (mm/L)
Group 1	Normal mice fed on balanced diet without EAC induction.	17.87±1.4	15.89±1.1	37.38±5	1.46±0.01	1.55±0.11
Group 2	Normal mice fed on balanced diet supplemented with milk fat CLA without EAC induction	18.12±1.6	16.11±1.2	37.51±4	1.49±0.02	1.82±0.14*
Group 3	TTM fed on normal diet (Cancer control)	22.78±1.5*	25.61±2*	137.92±9*	3.80±0.08*	1.04±0.05
Group 4	TTM fed on balanced diet supplemented with 40 mg/kg milk fat CLA	19.40±1.3**	16.20±1**	56.79±4**	2.24±0.02**	1.48±0.12**

The values expressed as mean±SE,

* Significant at P<0.05 as compared with normal control group (Group 1),

** Significant at P<0.05 as compared with TTM control group (Group 3). ALT=Aminotransferase, AST=Aspartate aminotransferase, ALP=Alkaline phosphatase, TAC=Total antioxidant capacity, CLA=Conjugated linoleic acid, TTM=Tumor transplanted mice, EAC=Ehrlich ascites carcinoma

#### Kidney function

Concerning the creatinine contents in the sera of the examined animal groups (Figure-5d), similar changes were detected by administration milk fat CLA either in normal mice groups or tumor transplanted ones. Creatinine level did not change in the sera of animals which were fed on diet containing 40 mg CLA/kg BW (G2) as compared to animals fed on a normal diet (G1) while, the level was significantly (p<0.05) elevated from 1.46 to 3.80 mg/DL by cancer cell transplantation (G3). The elevation in creatinine levels by cancer induction was significantly reduced to 2.24 mg/DL (Table-5 and Figure-5d) after feeding the TTM on a diet rich in CLA at a dose of 40 mg CLA/kg BW (G4). Therefore, diet supplemented with milk fat CLA at a level of 40 mg CLA/kg BW significantly improved the dangerous elevation in creatinine levels and can, in turn, protect the kidney from the negative effects of cancer.

#### Serum proteins

Data in Table-6 show the serum protein (total and A/G) results of normal and TTM fed on a normal diet and that supplemented with milk fat CLA. Total proteins (Figure-5a) and albumin (Figure-5b) are significantly (p<0.05) decreased, from 5.39 to 3.92 g/DL and from 2.85 to 1.41 g/DL, respectively, by cancer induction but the levels were significantly re-increased, to 5.15 g/DL and 2.64 g/DL, respectively, by daily oral administration of 40 mg CLA/kg BW (G4). Globulin, however, showed opposite behavior which was increased by cancer transplantation then reduced by milk fat CLA supplementation. A/G ratio was decreased as compared to normal mice.

**Table-6:** Effect of daily oral administration of milk fat rich in CLA on serum proteins (g/DL) in EAC inoculated mice.

Groups	Total	Albumin	Globulin	A/G ratio
Group 1	5.39±0.1	2.85±0.1	2.51±0.03	1.12±0.05
Group 2	5.74±0.2	3.01±0.2	2.73±0.02	1.10±0.04
Group 3	3.92±0.1*	1.41±0.2*	2.54±0.04	0.56±0.04*
Group 4	5.17±0.1	2.64±0.2**	2.52±0.02**	1.04±0.01**

The values expressed as mean±SE,

* Significant at (p*<*0*.*05) as compared with normal control group (Group 1),

** Significant at (p*<*0*.*05) as compared with TTM control group (Group 3). CLA=Conjugated linoleic acid, TTM=Tumor transplanted mice, EAC=Ehrlich ascites carcinoma

Liver being the immediate target organ affected by fast-growing EAC cells, uncontrolled gene expression. Patra *et al*. [39] stressed that serum enzymes are consider early indicators of neoplasia and as auxiliary for recognizing the extent of cancer progression or regression. Tumor in human or animals is known to affect the liver functions. Significant elevation in AST, ALT, and ALP activity levels in the sera of TTM indicate damage in liver cells, while elevation in creatinine level is due to kidney dysfunction [42]. Halaby *et al*. [43] stated that several investigators also reported liver damage and disturbances in hepatic cell metabolism in TTM with EAC cells, and the serum level of liver enzymes is considered as reliable indices of hepatotoxicity. Nearly similar findings were reported by Habib *et al*. [44] who reported that damage effects of EAC on the renal tissue were reflected by a significant increase in serum creatinine. Such biochemical change is the outcome of nephropathy.

**Figure-6:**
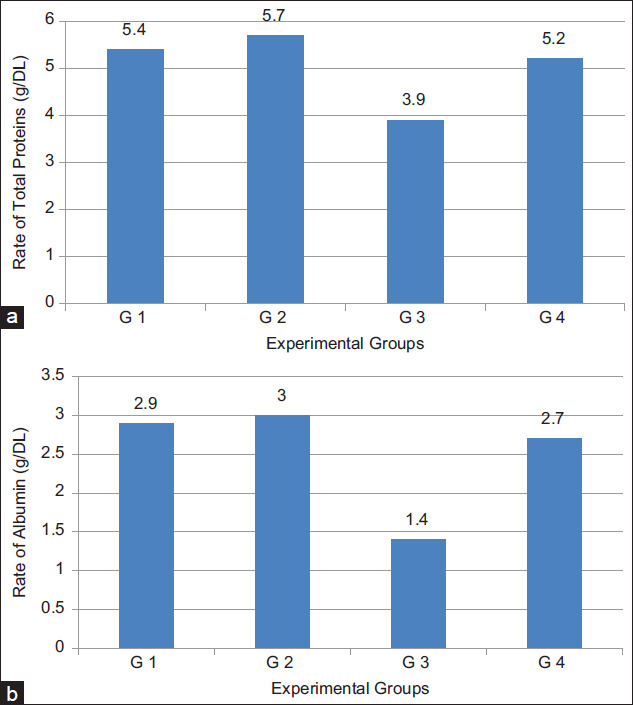
(a) Total proteins and (b) albumin in sera of Ehrlich ascites carcinoma inoculated mice after oral administration of milk fat rich in conjugated linoleic acid.

The treatment of cancer with natural anticancer agents can inhibit the bad effect in liver [40]. Our results showed that diet supplemented with milk fat CLA greatly improved the disturbances in liver enzymes by tumor transplantation. In a similar study on TTM with EAC, Al Abdan [45] reported that administration of a-lipoic acid (LA) regulated liver enzymes ALT, AST, ALP, and indicated the efficiency of LA as cancer inhibitor and its therapeutic influence. Lalithadevi *et al*. [35] declared that reduction of hepatic enzymes level in serum is one of the indications of the antitumor potential and treatment against tumor cells; they attributed the hepatoprotective effect of CLA to its isomers that induce cytotoxicity in hepatocytes or through its antioxidant property. The disturbance in liver and kidney function by tumor transplantation will consequently affect protein levels in blood of the experimental animals [46]. The significant decrease in total serum proteins and albumin, in the present study agrees with the results obtained by Saad *et al*. [46] who also mentioned that in mice treated with EAC cells, total proteins, albumin percentages, and A/G ratios were decreased as compared to normal mice. In our previous study, the results confirmed clear anticancer activity for milk rich in CLA in both tumor cell lines (*in vitro*) and TTM (*in vivo*). The study came to the conclusion that milk rich in CLA enhance cancer cells to enter the apoptotic pathway [47]. As shown in Table-6 and Figure-6, oral administration of supplemented diet with milk fat CLA to TTM, led to great disappearance for abnormalities in the biochemical parameters and returned them nearly to the normal levels. The reduced level of these biochemical parameters in serum is one of the indications of the antitumor potential and treatment against tumor cells [48].

The present work showed that cancer induction into mice gave changes (decreases) in the TAC, which was modified by milk fat CLA administration (Table-5). In this concern, we can suggest that CLA has a potential therapeutic complement in the cure or prevention of different disturbances associated with cancer due to imbalance in the cellular oxidoreductive status. This suggestion was previously confirmed by Al Abdan [45] who also suggested that supplementation with natural compounds that are thought to influence liver function can improve liver dysfunction in TTM, in addition to its oncostatic effect. The natural compounds as polyunsaturated fatty acids (PUFAs), including CLA, exhibit antioxidant activity and have a major role in cancer treatment. It is well documented that the higher the intracellular concentrations of lipid peroxides, the lower the mitotic rate and vice versa, tumor cells are resistant to lipid peroxidation in comparison with normal cells [35]. This observation is in tune with the fact that cancer cells have lower microsomal phospholipid content and low content of PUFAs [49]. Hence, accumulation of toxic lipid peroxides only in tumor but not in normal cells may be the cause of CLA tumoricidal action. Coadministration of these bioactive CLA isomers in conjunction with conventional anticancer drugs can induce remission of other tumors in humans. Thus, even drug-resistant cancers could be effectively treated using this bioactive lipid-based therapeutic approach [50].

## Histopathological results

### 

#### Liver

**Figure-7:**
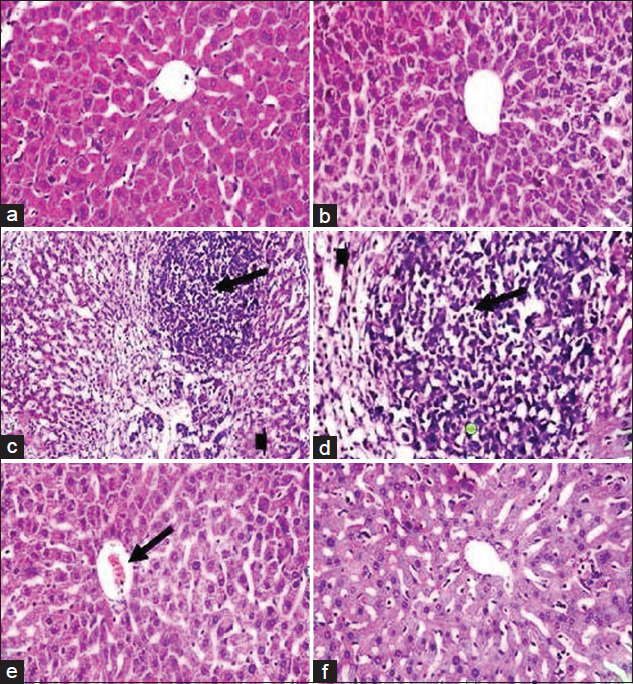
Photomicrograph of mice liver from Groups 1 and 2 (a and b) showing normal histological appearance of liver including central vein, blood sinusoids, hepatic cells, and Kupffer cells. Histological structure of mice liver from Group 3 (c and d) showing enlarged and congested central vein, degenerated hepatocytes (arrow head), infiltration of tumor cells, and leukocytes (arrow). Liver of mice from Group 4 (e) showing mild congested central vein (arrow). Mice liver from Group 5 (f) showing. Nearly normal histological appearance (H and E, 400×).

Both control negative mice group fed on a normal diet (G1) and the mice group fed on normal diet supplemented with milk fat CLA (G2) showed normal structure of liver, formed from polygonal lobules with indistinct outlines. Hepatocytes were polyhedral in shape, had vesicular spherical nuclei with prominent nucleoli and eosinophilic cytoplasm. Hepatocytes were arranged in cords that radiated out from the center of each lobule where the central vein situated. Between the hepatic cords hepatic sinusoids are localized and contained fine arrangement of Kupffer cells (Figure-7a and b), these findings were approved by Abou Zaid *et al*. [51]. EAC bearing mice positive group, (G3) revealed massive pathological alterations distributed throughout the hepatic tissue. Liver showed enlarged and congested central vein, coagulative degeneration was also obvious in the hepatocytes. Kupffer cells were abundant more than normal. Infiltration of cancer cells mixed with leukocytes as a sign of tumor metastasis in liver tissue was reported (Figure-7c and d). These findings were in agreement with Bhattacharyya *et al*. [52]. Examination of liver sections obtained from EAC mice treated with milk fat CLA, 40 mg CLA/kg BW, (G4) revealed reasonable ameliorations to a great extent, but the central vein was still mildly congested and enlarged (Figure-7e). Hepatotoxicity induced by EAC cells were nullified by the preventive effects of milk fat CLA supplementation in mice treated with milk fat CLA 28 days before experimental EAC inoculation (G5) and revealed normal histological lesions nearly similar to those of the control negative group (Figure-7f).

#### Kidney

**Figure-8:**
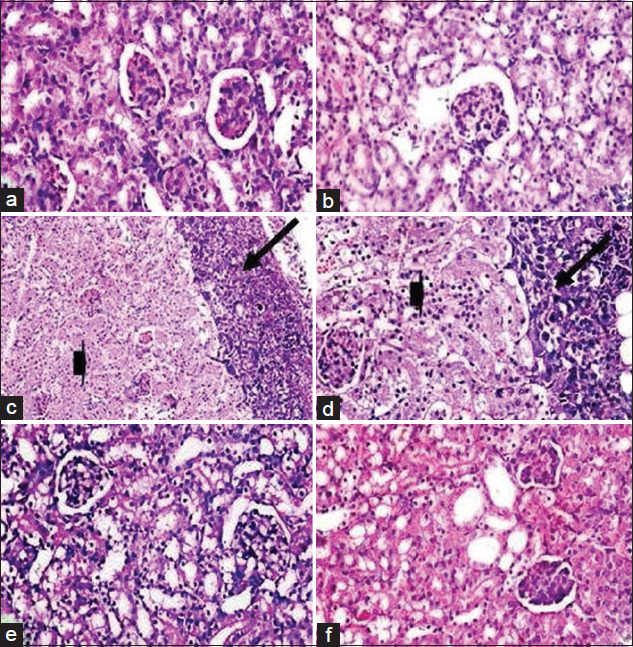
Photomicrograph of mice kidneys from Groups 1 and 2 (a and b) showing normal glomeruli and normal renal tubules. Histological structure of mice kidneys from Group 3 (c and d) showing glomerular atrophy and degenerated renal tubules (arrow head), proteinous casts in the lumen of renal tubules, and infiltration of tumor cells and leukocytes (arrow). Kidneys of mice from Group 4 (e) showing mildly degenerated renal tubules. Mice kidneys from Group 5 (f) showing nearly normal glomeruli and renal tubules, (H and E, 400×).

Both control negative mice group fed on a normal diet (G1) and mice group fed on normal diet supplemented with milk fat CLA (G2) were composed of two main regions; the renal cortex and medulla which possess normal histological features. The renal cortex is enclosed by numerous renal corpuscles, each made up of glomeruli and the Bowman’s capsule. There is a characteristic normal space between the glomeruli and Bowman’s capsule to allow renal filtration. Proximal and distal convoluted tubules surround the renal corpuscles; these tubules have inner wide luminal space lined externally with cuboidal epithelium (Figure-8a and b). Positive group, EAC bearing mice (G3) revealed marked damage of renal tissues which are represented in degenerated renal tubules and glomerular atrophy. Proteinous casts in the lumen of the renal tubules were also observed. Infiltration of tumor cells mixed with leukocytes as a sign of tumor metastasis in kidneys tissue was clearly denoted, (Figure-8c and d); this result is in concomitance with Abd El-Wahab and Fouda [53]. Examination of kidneys sections obtained from EAC mice treated with milk fat CLA, 40 mg CLA/kg BW (G4) revealed reasonable ameliorations to a great extent, with somewhat little degeneration in renal tubules (Figure-8e). Nephrotoxicity induced by EAC cells was nullified by the preventive effects of milk fat CLA supplementation in mice treated with milk fat CLA 28 days before EAC inoculation (G5) and revealed nearly normal histological lesions similar to those of the control negative group (Figure-8f).

CLA has long been widely present in various diets; it has a wide range of beneficial activities in human health due to the biological importance of its isomers [54]. Milk fat is the richest natural source of CLA with concentrations typically ranging between 4 and 5 mg/g fat [55]; in this study, we could obtain milk fat contain 20 mg/g fat. Consumption of foods naturally enriched with CLA during lifetime, can reduce the risk of several diseases. Such positive effects include anticarcinogenic, antiatherogenic, antidiabetic, antiobesity, and enhancement of immune system [18]. The effects seem to be mediated primarily by two CLA isomers: c9, t11 and t10, c12 but the impact may differ depending on the isomer [56]. The obtained results in this study agrees with the most human studies that reported no significant changes in liver functions, morphology, or signs of hepatic lipodystrophy after CLA administration [15]. Milk fat CLA has been shown *in vivo* (TTM) reduction in tumor growth; in accord with Field and Schley [57] who justified the reduction in tumor growth by slowing cell replication, cell death through apoptosis or both. Islam *et al*. [58] mentioned that apoptosis is one of the most common mechanisms of the anticarcinogenic CLA isomers in animal and human cancer cells, which is mediated by dysfunction of mitochondria. They added that isomers of CLA induce cytotoxicity in cancer cells without significantly affecting the normal cells.

The obtained decline in ascitic fluid volume in CLA treated mice is due to significant inhibition of cell proliferation (Figure-2b) and to enhance apoptosis in EAC cells, agrees with the result obtained by Dinicola *et al*. [59]. Ding and Nguyen [60] confirmed that antiproliferative activity of CLA isomers is dependent on cancer cells through suppression of the gap junctional intercellular communication (GJIC), which connect the cytoplasm of neighboring cells to maintain tissue homeostasis through allowing the passage of small cytoplasmic molecules and ions, and regulate cell proliferation and apoptosis. McLachlan *et al*. [61] explained that deficiencies in connexin genes (Cx), proteins, especially Cx43 which present in human breast tissue and most of body cells, act as a cancer marker. They added that, in cancer cells CLA isomers can enhance GJIC by inducing Cx43 gene expression, and this is an indication of its anticancer action; according to several studies [62,63] confirmed that enhancing GJIC through Cx43 upregulation in tumor cells is positively correlated with anticancer activities. Rakib *et al*. [64] also demonstrated the downregulation inhibition of GJIC in human MCF-10A cells by CLA isomers which protect the phosphorylation of Cx43 and maintain GJIC. Song *et al*. [65] in another study reported that apoptosis in cancer cells (MCF-7) was associated with upregulation of GJIC through Cx43 expression mediated by inactivation of the activity of the nuclear factor-kB activity.

Low concentrations of estrogen can downregulate Cx43 expression and lead to instability of the Cx43 protein in many cancer cells. CLA display the estrogen antagonistic activities through the inhibition of the ERa-mediated signaling in ER-positive MCF-7 human breast cancer cells; unlike MCF-7 cells, the inhibitory effects of CLA on cell growth were weak or lost in ER-negative MDA-MB-231 breast cancer cells. Furthermore, CLA triggers ERa/protein phosphatase 2 (PP2A) complex formation and increase PP2A activity, and this explains the decreased ERa-ERE binding activity by CLA supplementation [23]. Hence, Amaru *et al*. [24] proved that CLA effectively reduces breast cancer risk by inhibiting breast tumor initiation, promotion, and progression.

Our present study showed strong anticancer activity and confirmed several studies obtained similar results after treatment with CLA. There is a significant interest for using milk fat CLA as an adjuvant agent in combination with chemotherapy drugs.

## Conclusion

In the light of the above observations, it can be concluded that these novel results *in vivo* studies indicate the significant promising anticancer activity of milk fat CLA which may be due to the additive and synergistic activity of its natural isomers. The activity was confirmed by significant improvement of MST and decrease of WBC count. On the other hand, milk fat CLA can also be used as adjuvant therapy in combination with the existing anticancer drugs. Further investigation to learn more about the mechanism of this antitumor activity are needed so that it can be formulated and be tried clinically.

## Authors’ Contributions

AMA: Conceptualized and designed the research. AhMA, DGA, SSA, and MHB: Participated in material preparation. AMA, DGA, andAhMA: Designed the *in vivo* experiment. DGA and SAA: Performed the hematological profile. AhMA, and MAA: performed the biochemical analysis. SSA performed the histopathology. SAA and MHB: Contributed in coordinating the data collections. AMA, DGA, AhMA, and SAA: Wrote the first draft of the manuscript. GE and AAM: Criticized and revised the text according to its scientific content. DGA: Conducted the statistical analysis of results, revised, and finalized the manuscript for submission. RAA: Performed editing and proofreading of the manuscript. All authors read and approved the final version of the manuscript.

## References

[1] Meera R, Chidambaranathan N (2017). Anticancer activity of ethanolic extract of *Crataeva maqua* Lour (DC) against EAC cell lines in mice. J. Pharm. Sci. Res.

[2] Babasaheb P.B, Shrikant S.G, Ragini G.B, Jalinder V.T, Chandrahas N.K (2010). Synthesis and biological evaluation of simple methoxylated chalcones as anticancer, anti-inflammatory and antioxidant agents. Bioorg. Med. Chem.

[3] World Health Organization. Cancer (2018). Fact sheets, 12 September 2018.

[4] Burney I.A, Furrukh M, Al-Moundhri M.S (2014). What are our options in the fight against breast cancer?. Sultan Qaboos Univ Med. J.

[5] Samiron S, Sohel R, Shakila R, Mahmudul H, Sadiur R.S, Atikullah S.M, Mushiur R, Tanjir I.H, Ikramul K, Protic J, Niaz M, Shovon A, Arman A (2018). Evaluation of anticancer properties against ehrlich ascites carcinoma (EAC) cell line, cytotoxic and analgesic activity of methanol extract of *Hibiscus moscheutos*in Swiss Albino mice. Int. J. Pharm. Sci. Rev Res.

[6] Moorthi C, Manavalan R, Kathiresan K (2011). Nanotherapeutics to overcome conventional cancer chemotherapy limitations. J. Pharm. Pharm. Sci.

[7] Kaur R, Kapoor K, Kaur H (2011). Plants as a source of anticancer agents. J. Nat. Prod. Plant Resour.

[8] Arab A, Akbarian S.A, Ghiyasvand R, Miraghajani M (2016). The effects of conjugated linoleic acids on breast cancer:A systematic review. Adv. Biomed. Res.

[9] Huang S.S, Chiu C.S, Lin T.H, Lee M.M, Lee C.Y, Chang S.J, Hou W.C, Huang G.J, Deng J.S (2013). Antioxidant and anti-inflammatory activities of aqueous extract of *Centipeda minima*. J. Ethnopharmacol.

[10] Mills S, Ross R.P, Hill C, Fitzgerald G.F, Stanton C (2011). Milk intelligence:Mining milk for bioactive substances associated with human health. Int. Dairy J.

[11] Ozaslan M, Karagoz I.D, Kilic I.H, Muhammed E.G (2011). Ehrlich ascites carcinoma. Afr. J. Biotechnol.

[12] Kabel A.M, Abdel-Rahman M.N, El-Sisi A.E.D, Haleem M.S, Ezzat N.M, El Rashidy M.A (2013). Effect of atorvastatin and methotrexate on solid Ehrlich tumor. Eur. J. Pharmacol.

[13] Badr M.O, Edrees N.M, Abdalla A.A, Nasr El-Deen A.M.N, Neamat-Alla A.N.F, Ismail H.T (2011). Anti-tumor effects of Egyptian propolis on Ehrlich ascites carcinoma. Vet. Ital.

[14] Yuan G.F, Chen X.E, Li D (2014). Conjugated linoleic acids and their bioactivities:A review. Food Funct.

[15] Dilzer A, Park Y (2012). Implication of conjugated linoleic acid (CLA) in human health. Crit. Rev. Food Sci. Nutr.

[16] Martínez-Monteagudo S.I, Saldaña M.D.A (2015). Retention of bioactive lipids in heated milk:Experimental and modelling. Food Bioprod. Process.

[17] Gorissen L, Leroy F, De Vuyst L, De Smet S, Raes K (2015). Bacterial production of conjugated linoleic and linolenic acid in foods:A technological challenge. Crit. Rev. Food Sci. Nutr.

[18] Chen S.C, Lin Y.H, Huang H.P, Hsu W.L, Houng J.Y, Huang C.K (2012). Effect of conjugated linoleic acid supplementation on weight loss and body fat composition in a Chinese population. Nutrition.

[19] Al-Safi Z.A, Santoro N (2014). Menopausal hormone therapy and menopausal symptoms. Fertil. Steril.

[20] Rozenberg S, Vandromme J, Antoine C (2013). Postmenopausal hormone therapy:Risks and benefits. Nat. Rev. Endocrinol.

[21] Munsell M.F, Sprague B.L, Berry D.A, Chisholm G, Trentham-Dietz A (2014). Body mass index and breast cancer risk according to postmenopausal estrogen-progestin use and hormone receptor status. Epidemiol. Rev.

[22] Wang J, Liu X, Zhang X, Liu J, Ye S, Xiao S, Chen H, Wang H (2013). Induction of apoptosis by c9, t11-CLA in human endometrial cancer RL 95-2 cells via ERalpha-mediated pathway. Chem. Phys. Lipids.

[23] Bocca C, Bozzo F, Cannito S, Colombatto S, Miglietta A (2010). CLA reduces breast cancer cell growth and invasion through ERalpha and PI3K/Akt pathways. Chem. Biol. Interact.

[24] Amaru D.L, Biondo P.D, Field C.J (2010). The role of conjugated linoleic acid in breast cancer growth and development. Open Nutraceuticals J.

[25] Benjamin S, Prakasan P, Sreedharan S, Andre-Denis G, Wright A.G, Spener F (2015). Pros and cons of CLA consumption:An insight from clinical evidences. Nutr. Metab.

[26] Zahan R, Alam M.B, Islam M.S, Sarker G.C, Chowdhury N.C, Hosain S.B, Mosaddik M.A, Jesmin M, Haque M.E (2011). Activity of *Alangium salvifolium* flower in ehrlich ascites carcinoma bearing mice. Int. J. Cancer Res.

[27] Samudrala P.K, Augustine B.B, Kasala E.R, Bodduluru L.N, Barua C, Lahkar M (2015). Evaluation of antitumor activity and antioxidant status of *Alternanthera brasiliana* against Ehrlich ascites carcinoma in Swiss albino mice. Pharmacogn. Res.

[28] Abu Osman M, Rashid M.M, Abdul Aziz M, Habib M.R, Karim M.R (2011). Inhibition of Ehrlich ascites carcinoma by *Manilkara zapota* Lstem bark in Swiss albino mice. Asian Pac. J. Trop. Biomed.

[29] García-Moreno E, Tomás A, Atrián-Blasco E, Gascón S, Romanos E, Rodriguez-Yoldi M, Cerradaa E, Laguna M (2016). *In vitro* and *in vivo* evaluation of organometallic gold(I) derivatives as anticancer agents. Dalton Trans.

[30] Sangameswaran B, Pawar S.P, Saluja M.S, Sharma A (2012). Antitumor activity of *Sida veronicaefolia* against Ehrlich ascites carcinoma in mice. J. Pharm. Res.

[31] Fernands D.P, Pimentel M.L, Do Santos F.A, Praxedes E.A, De Brito P.D, Lima M.A, Lelis I.C, De Macedo M.F, Bezerra M.B (2018). Hematological and biochemical profile of BALB/c nude and C57BL/6 SCID female mice after ovarian xenograft. An. Acad. Bras. Cienc.

[32] Santos E.W, Oliveira D.C, Hastreiter A, Silva G.B, Beltran J.S, Tsujita M, Crisma A.R, Neves S.M, Fock R.A, Borelli P (2016). Hematological and biochemical reference values for C57BL/6, Swiss Webster and BALB/c mice. Braz. J. Vet. Res. Anim. Sci.

[33] Nader F, Hanyath A.R, Wittwer C.T Tietz Fundamentals of Clinical Chemistry and Molecular Diagnostics.

[34] Moreira V.G, Vaktangova N.B, Gago M.M, Gonzalez B.L, Alonso S.G, Rodriguez E.F (2018). Overestimation of albumin measured by bromocresol green vs bromocresol purple method:Influence of acute-phase globulins. Lab. Med.

[35] Lalithadevi B, Muthiah N.S, Murty K.S.N (2018). Antioxidant activity of conjugated linoleic acid. Asian J. Pharm. Clin. Res.

[36] Bancroft J.D, Suvarna K, Layton C (2012). Bancroft's Theory and Practice of Histological Techniques.

[37] Jemal A, Bray F, Center M.M, Ferlay J, Ward E (2011). Global cancer statistics. CA Cancer J.Clin.

[38] Reddy V.N, Nagarathna P.K.M, Divya M (2013). Evaluation of anti-cancer activity of *Ruellia tuberosa* on EAC induced mammary tumor. Int. J. Pharmacol. Toxicol.

[39] Patra S, Muthuraman M.S, Prabhu A.R, Priyadharshini R.R, Parthiban S (2015). Evaluation of antitumor and antioxidant activity of S*argassum tenerrimum*against Ehrlich ascites carcinoma in mice. Asian Pac. J. Cancer Prev.

[40] Hashem M.A, Mahmoud E.A, Abd-Allah N.A (2020). Alterations in hematological and biochemical parameters and DNA status in mice bearing Ehrlich ascites carcinoma cells and treated with cisplatin and cyclophosphamide. Compar. Clin. Pathol.

[41] Etim E.A, Adebayo Y.A, Ifeanyi O.E (2018). Effect of *Luffa cylindrica* leaf extract on hematological parameters of Swiss Albino mice. Med. Aromat. Plants.

[42] Dolai N, Karmakar I, Kumar R.B.S, Kar B, Bala A, Haldar P.K (2012). Evaluation of antitumor activity and *in vivo* antioxidant status of *Anthocephalus cadamba* on Ehrlich ascites carcinoma treated mice. J. Ethnopharmacol.

[43] Halaby M.S, Farag M.H, Mahmoud S.A.A (2015). Protective and curative effect of garden cress seeds on acute renal failure in male albino rats. Middle East J. Appl. Sci.

[44] Habib M.R, Aziz M.A, Karim M.R (2010). Inhibition of Ehrlich's ascites carcinoma by ethyl acetate extract from the flower of *Calotropis gigantea* L in mice. J. Appl. Biomed.

[45] Al Abdan M (2012). Alfa-lipoic acid controls tumor growth and modulates hepatic redox state in ehrlich-ascites-carcinoma-bearing mice. 2012. Sci. World J.

[46] Saad E.A, Hassanien M.M, El-mezayen H.A, ELmenawy N.M (2017). Regression of murine Ehrlich ascites carcinoma using synthesized cobalt complex. Med. Chem. Commun.

[47] Abd El-Gawad A.M, Aboul-Enein A.M, Ali R.A, Abo El-Hassan D.G, Bakr M.H (2020). The Anticancer Activity of Milk Rich in Conjugated Linoleic Acid, CLA (Under Publication).

[48] Ali D.A, Badr N.K, Abou-El-Magd R.F (2015). Antioxidant and hepatoprotective activities of grape seeds and skin against Ehrlich solid tumor induced oxidative stress in mice. Egypt. J. Basic Appl. Sci.

[49] Das U.N, Madhavi N (2011). Effect of polyunsaturated fatty acids on drug-sensitive and resistant tumor cells *in vitro*. Lipids Health Dis.

[50] Das U.N (2020). Immune system inflammation, and essential fatty acids and their metabolites in cancer. In:Molecular Biochemical Aspects of Cancer. Humana, New York.

[51] Abou Zaid O.A.R, Hassanein M.R.R, El-Senosi Y.A, El-Shiekha M.F (2011). Ameliorative effect of curcumin and tannic acid on tumor-induced in female mice. Benha Vet. Med. J.

[52] Bhattacharyya A, Mandal D, Lahiry L, Bhattacharyya S, Chattopadhyay S, Ghosh U.K, Sa G, Das T (2007). Black tea-induced amelioration of hepatic oxidative stress through antioxidative activity in EAC-bearing mice. J. Environ. Pathol. Toxicol. Oncol.

[53] Abd El-Wahab S.M, Fouda F.M (2009). Histological and histochemical study on the effect of Ehrlich ascites carcinoma on the liver and kidney of mice and the possible protective role of tetrodotoxin. Egypt. J. Biol.

[54] Rishehri S.M.D, Rahbar A.R, Ostovar A (2019). Effects of conjugated linoleic acid intake in the form of dietary supplement or enriched food on c-reactive protein and lipoprotein (a) levels in humans:A literature review and meta-analysis. Iran. J. Med. Sci.

[55] Marín M.P, Meléndez P.G, Aranda P, Ríos C (2018). Conjugated linoleic acid content and fatty acids profile of milk from grazing dairy cows in southern Chile fed varying amounts of concentrate. J. Appl. Anim. Res.

[56] Korhonen H.J (2011). Bioactive milk proteins peptides and lipids and other functional components derived from milk and bovine colostrum. In:Functional Foods.

[57] Field C.J, Schley P.D (2004). Evidence for potential mechanisms for the effect of conjugated linoleic acid on tumor metabolism and immune function. Am. J. Clin. Nutr.

[58] Islam M.A, Kim Y.S, Oh T.W, Kim G.S, Won C.K, Hoon G, Kim H.G, Choi M.S, Kim J.O, Ha Y.L (2010). Superior anticarcinogenic activity of trans, trans-conjugated linoleic acid in Nmethyl-N-nitrosourea-induced rat mammary tumorigenesis. J. Agric. Food Chem.

[59] Dinicola S, Cucina A, Pasqualato A, Proietti S, D'Anselmi F (2010). Apoptosis-inducing factor and caspase-dependent apoptotic pathways triggered by different grape seed extracts on human colon cancer cell line Caco-2. Br. J. Nutr.

[60] Ding Y, Nguyen T.A (2012). Gap junction enhancer potentiates cytotoxicity of cisplatin in breast cancer cells. J. Cancer Sci. Ther.

[61] McLachlan E, Shao Q, Laird D.W (2007). Connexins and gap junctions in mammary gland development and breast cancer progression. J. Membr. Biol.

[62] Zahran M, Aboul-Enein A, Aboll-Ella F (2005). Molecular changes on cancer cells as affected by willow extracts. Res. J. Agric. Biol. Sci.

[63] Tishchenko A, Azorín D.D, Vidal-Brime L, Muñoz M.J, Arenas P.J, Pearce C, Girao H, Ramón Y, Cajal S, Aasen T (2020). Cx43 and associated cell signaling pathways regulate tunneling nanotubes in breast cancer cells. Cancers.

[64] Rakib M.A, Kim Y.S, Jang W.J, Jang J.S, Kang S.J, Ha Y.L (2011). Preventive effect of t,t-conjugated linoleic acid on 12-O- tetradecanoylphorbol-13-acetate-induced inhibition of gap junctional intercellular communication in human mammary epithelial MCF-10A cells. J. Agric. Food Chem.

[65] Song H.J, Sneddon A.A, Heys S.D, Wahle K.W.J (2006). Induction of apoptosis and inhibition of NF-??B activation in human prostate cancer cells by the cis-9, trans-11 but not the trans-10, cis-12 isomer of conjugated linoleic acid. Prostate.

